# Selfie Aging Index: An Index for the Self-assessment of Healthy and Active Aging

**DOI:** 10.3389/fmed.2017.00236

**Published:** 2017-12-22

**Authors:** Judite Gonçalves, Maria Isabel Gomes, Miguel Fonseca, Tomás Teodoro, Pedro Pita Barros, Maria-Amália Botelho

**Affiliations:** ^1^Nova Healthcare Initiative Research, Nova School of Business and Economics, Universidade Nova de Lisboa, Lisbon, Portugal; ^2^School of Business and Management, Queen Mary University of London, London, United Kingdom; ^3^Center for Mathematics and Applications, Faculty of Sciences and Technology, Universidade Nova de Lisboa, Lisbon, Portugal; ^4^Chronic Diseases Research Center, Nova Medical School, Universidade Nova de Lisboa, Lisbon, Portugal

**Keywords:** Selfie Aging Index, healthy aging, active aging, aging index, multidimensional index, biopsychosocial assessment model, ordered probit model, self-assessed health

## Abstract

**Introduction:**

Governments across Europe want to promote healthy and active aging, as a matter of both public health and economic sustainability. Designing policies focused on the most vulnerable groups requires information at the individual level. However, a measure of healthy and active aging at the individual level does not yet exist.

**Objectives:**

This paper develops the *Selfie Aging Index* (SAI), an individual-level index of healthy and active aging. The SAI is developed thinking about a tool that would allow each person to take a *selfie* of her aging status. Therefore, it is based entirely on self-assessed indicators. This paper also illustrates how the SAI may look like in practice.

**Methods:**

The SAI is based on the Biopsychosocial Assessment Model (MAB), a tool for the multidimensional assessment of older adults along three domains: biological, psychological, and social. Indicators are selected and their weights determined based on an ordered probit model that relates the MAB indicators to self-assessed health, which proxies healthy and active aging. The ordered probit model predicts the SAI based on the estimated parameters. Finally, predictions are rescaled to the 0–1 interval. Data for the SAI development come from the Study of the Aging Profiles of the Portuguese Population and the Survey of Health, Aging, and Retirement in Europe.

**Results:**

The selected indicators are BMI, having difficulties moving around indoors and performing the activities of daily living, feeling depressed, feeling nervous, lacking energy, time awareness score, marital status, having someone to confide in, education, type of job, exercise, and smoking status. The model also determines their weights.

**Conclusion:**

Results shed light on various factors that contribute significantly to healthy and active aging. Two examples are mental health and exercise, which deserve more attention from individuals themselves, health-care professionals, and public health policy. The SAI has the potential to put the individual at the center of the healthy and active aging discussion, contribute to patient empowerment, and promote patient-centered care. It can become a useful instrument to monitor healthy and active aging for different actors, including individuals themselves, health-care professionals, and policy makers.

I have enjoyed greatly the second blooming … suddenly you find – at the age of 50, say – that a whole new life has opened before you.

Agatha Christie

## Introduction

In 2016, people aged 55 and over (55+) accounted for 32% of the European Union’s (EU) population, and people aged 65 and over (65+) for 19%. The EU countries with the oldest populations are Italy, Germany, Greece, and Portugal, where the 55+ represent 34–35% of the population and the 65+ represent 21–22% (own calculations based on Eurostat data). These numbers are expected to rise in the future, challenging EU governments to promote the healthy and active aging of their populations, as a matter of both public health and economic sustainability.

The concept of healthy and active aging was defined by the World Health Organization (WHO) as the process of optimizing opportunities for health to enhance quality of life as people age. The word “healthy” refers to physical, mental, and social well-being, while the word “active” refers to continuing participation in social, economic, cultural, spiritual, and civic affairs (1). It should be noted that some studies focus specifically on healthy aging or active aging alone. Other studies refer only to healthy aging or active aging but possibly mean both healthy and active aging.

To design policies to promote healthy and active aging, as well as to track their progress, measurement is crucial. With that in mind, the European Commission (EC) and the United Nations Economic Commission for Europe introduced the Active Aging Index (AAI) in 2012. The AAI is a multidimensional index that measures the level to which older people (55+) live independent lives, participate in the labor market and social activities as well as their capacity to actively age (2). As its name suggests, the AAI focuses specifically on active aging. It conceptualizes active aging as “the situation where people continue to participate in the formal labor market as well as engage in other unpaid productive activities and live healthy, independent, and secure lives as they age” (3). The AAI represents the first initiative to measure healthy and active aging. It has inspired the measurement of healthy and active aging around the world, which clearly shows the increasing interest in the aging phenomenon (4, 5).

The AAI provides a societal perspective of the aging phenomenon and is a useful tool for top-down policy design. However, it only allows for the comparison of average levels of active aging across countries. Designing policies focused on the most vulnerable groups requires information about the distributions of healthy and active aging within countries, as well as information about how healthy and active aging correlates with individual characteristics. To obtain such information, we need to measure healthy and active aging at the individual level. To that end, two recent studies develop individual-level indices of active aging based on the AAI conceptual framework (6, 7).

It is not clear that the AAI conceptual framework, developed to capture active aging at the aggregate level, is appropriate to measure active aging at the individual level. The four domains of the AAI are (1) employment, (2) participation in society, (3) independent, healthy, and secure living, and (4) capacity and enabling environment for active aging. At the individual level, these domains include indicators such as (1) being employed, (2) looking after grandchildren, (3) worrying about vandalism and crime, and (4) Internet use (7). Such indicators do not appear relevant for example in a study about older people’s perceptions of active aging. According to that study, individuals associate active aging with maintaining physical health and functioning, leisure and social activities, mental functioning and activity, and social relationships and contacts (8). However, this apparent discrepancy does not imply that the AAI conceptual framework is inappropriate for an individual-level index. In fact, such discrepancy may simply reflect different perspectives of older people—the “insiders”—and researchers—the “outsiders”— as has been documented [e.g., Ref. (9)].

The AAI-based individual-level indices have other potential limitations. First, the expert group who developed the AAI chose the weights of the indicators, which implies value judgments. Second, in the individual-level indices all indicators are dichotomized. Such strategy does not take full advantage of the variations in the data.

Focusing specifically on healthy aging, Lara et al. (10) provide a list of measures that capture key features of healthy aging, grouped into five domains: physiological and metabolic health, physical capability, cognitive function, social well-being, and psychological well-being (e.g., cardiovascular function, strength, episodic memory, mental health, perceived social support). Kuh et al. (11) conceptualize healthy aging within a life-course framework, distinguishing between healthy biological aging and changes in psychological and social well-being, and provide a review of objective measures of physical capability (e.g., grip strength, walking speed). To our knowledge, healthy aging has not been operationalized in any multidimensional index.

This paper develops the *Selfie Aging Index* (SAI). The SAI is based on a conceptual framework that tries to capture both healthy and active aging at the individual level. Its underlying methodology allows the weights to be determined by the variations and correlations present in the data, avoiding value judgments. The SAI is also innovative in that it may be entirely self-assessed, thinking about future applications of the SAI as a tool for older people to track their aging status. Thus, clinical indicators or measurements that require unusual tools are not considered (e.g., cardiovascular function, grip strength). Finally, this paper also illustrates how the SAI can be used in practice. To the absolute value of the SAI, which allows individuals to keep track of their aging status over time, we add a relative component that allows individuals to compare themselves to their peers.

## Materials and Methods

### Conceptual Framework

The SAI is based on the Biopsychosocial Assessment Model [MAB—*Modelo de Avaliação Biopsicosocial*, registered copyright No 4065/2007 (12)]. The MAB is a tool for the multidimensional assessment of older adults. It consists of several indicators grouped into three domains, for example ability to conduct daily activities such as bathing or eating (biological domain), feelings of depression or nervousness (psychological domain), and having someone to confide in (social domain). The full list of indicators is available in Table S1 in Supplementary Material. The domains and subdomains of the MAB capture the various elements of the conceptual definition of healthy and active aging of the WHO (see Introduction). Thus, the MAB seems suitable as a starting point to operationalize the measurement of healthy and active aging at the individual level. The MAB has been extensively validated and is currently used by the National Network of Continued Integrated Care (RNCCI—*Rede Nacional de Cuidados Continuados Integrados*) to characterize inpatients of the Portuguese health-care system (13). Unlike previous conceptual models, the MAB does not rely on clinical indicators or measurements that require unusual tools. This is appealing when we think about potential applications of the SAI as a tool for individuals to self-assess their aging status.

### Statistical Method

To construct the SAI, we need to select a subset of the MAB indicators. The original MAB includes a long list of indicators (see Table S1 in Supplementary Material), which is incompatible with a simple self-assessment tool for older adults. We also need to determine the weights for the aggregation of the indicators into a single index. Estimating an ordered probit model allows us to do both things, while avoiding value judgments in the selection of indicators and their respective weights. Indicators are selected based on their statistical significance and contribution to model fit, and the weights are determined based on correlations between the indicators. Another advantage of the ordered probit model is that it easily deals with any type of indicator, continuous or categorical. Ordered probit models have been used to construct health indices, in order to have a single continuous variable to measure health status [e.g., Ref. (14)].^1^ The idea behind this method is predicting a latent variable—the SAI—based on the MAB indicators. To do this, we use self-assessed health in the left hand-side of the ordered probit model [i.e., self-assessed health works as a kind of proxy for healthy and active aging, following, for example, Ref. (15, 16)]. The MAB indicators appear as right hand-side variables. Though self-assessed health consists in individuals’ ratings of their health in a scale (e.g., very bad, bad, fair, good, very good), the ordered probit model predicts a continuous variable based on the estimated parameters. The last step in computing the SAI is rescaling that predicted variable to a more intuitive 0–1 interval. This is done using the theoretical limits of the latent variable, which can be computed as the predicted values when all MAB indicators are set at their least and most favorable values. In sum, the SAI score is obtained by plugging the individual’s characteristics in the ordered probit model, obtaining the prediction in the latent scale, and rescaling it to the 0–1 interval.

### Data

We use data from the Study of the Aging Profiles of the Portuguese Population [EPEPP—*Estudo do Perfil de Envelhecimento da População Portuguesa* (17–19)] and the Survey of Health, Aging and Retirement in Europe (SHARE; Wave 4 (20)). The EPEPP was conducted specifically to evaluate the aging status of Portuguese older adults based on an earlier version of the MAB. It was designed to be representative of the Portuguese population aged 55+ and covered about 2,700 individuals, interviewed in 2005–2006. The SHARE is a broader survey of European older adults that also contains most of the MAB indicators. It was designed to be representative of the 50+ population, but we only consider individuals aged 55+. The SHARE includes about 1,700 individuals aged 55+ in Portugal, interviewed in 2011.

There are some differences between the two datasets in self-assessed health and the MAB indicators. Self-assessed health has four levels in the EPEPP survey (bad/very bad, poor, fair, good/very good), and five levels in the SHARE survey (poor, fair, good, very good, excellent). In the EPEPP, health complaints are classified into complaints that affect or not mobility, complaints regarding eyesight, and complaints regarding hearing. In the SHARE, the long list of health conditions available allows for a much more detailed analysis of health complaints, including for example complaints about the musculoskeletal system or the respiratory system. A few MAB indicators are lacking in each dataset. The EPEPP does not include indicators of lack of interest or trouble sleeping, and the SHARE does not include waist measurement, variables related with falls, measures of mobility outdoors or climbing stairs, spatial awareness, or time alone in a 24-h period. Table S1 in Supplementary Material provides the link between the MAB indicators and the questions in the EPEPP and SHARE.^2^

### Procedure

Given the differences between the EPEPP and SHARE surveys outlined above, the analyses of the two datasets are complementary. Estimating separate ordered probit models allows us to determine if the same MAB indicators appear relevant for healthy and active aging in both samples, providing external validity to our methodology. This is especially informative given that the EPEPP was conducted prior to the financial crisis and the SHARE was conducted after the crisis, i.e., the individuals in each sample faced different socioeconomic environments. Estimating separate models is the first stage in our procedure. All MAB indicators available in each survey are used. The original coding of the answers is maintained, in order to take full advantage of the information available. For example, the question “Over the past month, did you feel sad or depressed?” has four possible answers in the EPEPP survey (“No,” “Little time,” “Half of the time,” and “Most of the time”), and two possible answers in the SHARE survey (“Yes” and “No”).

In the second stage, the two datasets are combined. All MAB indicators available in both surveys are considered, except for health complaints (including complaints about the emotional status) because the corresponding questions are not posed in comparable ways (see Data and Table S1 in Supplementary Material). Combining the datasets requires recoding the answers to the questions that are posed in different ways in the two surveys. The new self-assessed health variable has three levels: poor/bad/very bad, fair, good/very good/excellent. Table S2 in Supplementary Material presents the new coding for all variables. Combining the datasets increases the sample size substantially, enhancing statistical power.

In the third stage, the model estimated in the combined dataset is simplified by eliminating the MAB indicators that do not contribute to improve model fit. To do this, the indicators are progressively added to the model according to their contribution to the log-likelihood (i.e., stepwise regression). With each addition, we assess the likelihood ratio test, as well as Akaike’s and the Bayesian Information Criteria (AIC, BIC). We consider that an indicator does not contribute to improve model fit if the null hypothesis of the likelihood ratio test is not rejected at the 10% significance level and the value of the AIC or the BIC increases.^3^ The SAI is calculated based on the resulting simplified model.

Recoding the variables when combining the two datasets may introduce bias. Furthermore, the EPEPP and SHARE surveys were conducted several years apart under different socioeconomic environments, which may affect the relationships between the variables. To explore these potential issues, we estimate an ordered probit model, including interactions between the MAB indicators and a binary indicator of the observation’s source (EPEPP or SHARE). Checking whether the associations between the MAB indicators and self-assessed health differ according to the source of data gives an indication of the extent of the problem. Given that one of the recoded variables is self-assessed health, we also test whether the estimated cutoffs significantly differ according to the dataset.

### Validity Checks

To investigate the validity of the SAI, we assess its value as a predictor of several outcomes, namely the probability of having had a doctor visit and number of doctor visits over the previous 12 months, mental health (EURO-D depression scale), number of chronic conditions, and number of symptoms. The EURO-D depression scale varies between 0 (not depressed) and 12 (very depressed) and is essentially a count of 12 items, including for example feeling guilty about anything, having no hopes for the future, and lacking appetite. The number of chronic conditions is a count of up to ten conditions including for example hypertension, diabetes, and arthritis. The number of symptoms is a count of up to 12 symptoms including for example pain, incontinence, and fatigue. None of these outcomes is an indicator in the SAI. We regress each outcome on the SAI and evaluate the statistical significance of the associated coefficient as well as the *R*^2^. The probability of having a doctor visit and the number of doctor visits are available in both surveys, but the remaining outcomes are available only in the SHARE.

### The SAI in Practice

In order to illustrate how the SAI may look like in practice, we calculate SAI scores for several hypothetical individuals. The absolute value of the SAI may be used by individuals to monitor their aging status over time. To provide some context, we add a relative component that allows individuals to compare themselves to their peers. The individual SAI scores are displayed in the distribution of SAI scores of people aged ±2 years. The larger sample size of the combined dataset allows us to have a significantly larger pool of peers for each individual in the dataset.

## Results

### Estimation Results Using the EPEPP Sample

Results based on the EPEPP sample are presented in Table S3 in Supplementary Material. The MAB indicators with a statistically significant coefficient, at the 10% significance level or lower, are health complaints, obesity (captured by waist measurement), having fallen due to internal causes, such as fainting, having sequelae from falling that affect mobility, needing someone’s help to perform the activities of daily living (ADLs), self-assessed emotional status, feeling depressed, feeling nervous, lacking energy, spending less than 8 h per day alone, having someone to confide in, exercising, and smoking status. Most of these variables have the expected associations with self-assessed health (i.e., the respective estimated coefficients have the expected signs). The remaining variables have no significant associations with self-assessed health. Looking at the magnitudes of the estimated coefficients, we can see that the variables with the largest associations with self-assessed health are complaints that do not affect mobility, self-assessed emotional status, feeling depressed most of the time, exercising more than 4 h per week, and smoking at present (coefficients larger than 0.3 in absolute value).

### Estimation Results Using the SHARE Sample

Table S4 in Supplementary Material presents the results based on the SHARE sample. The MAB indicators that have a statistically significant association with self-assessed health are health complaints except for those regarding the urinary system, difficulties moving around indoors and performing the ADLs, being depressed, being nervous, having trouble sleeping, lacking energy, marital status, living with someone else, having someone to confide in, education, and exercising. All of these variables except for living with someone else have the expected associations with self-assessed health. The coefficients of the remaining variables are not statistically significant. The variables with the largest associations with self-assessed health are complaints about hearing, the circulatory system, and the musculoskeletal system, having difficulties moving around indoors, and being divorced.

### Estimation Results Using the Combined Sample

Table 1 presents the results based on the combined dataset. Model 1 includes all MAB indicators available in both datasets. Model 2 excludes the indicators that do not contribute to improve the overall fit of the model. The excluded indicators are difficulties performing the instrumental activities of daily living (IADLs) and living with someone else, as well as age and gender. Including these variables in the model does not significantly increase the log-likelihood and the value of the AIC or the BIC increases, indicating worse fit (Table S5 in Supplementary Material).

**Table 1:** Estimation results of the ordered probit model using the combined sample.

	Model 1	Model 2
	Coefficient (SE)	Coefficient (SE)
Gender: female	−0.072 (0.048)	
Age	−0.079** (0.036)	
Age^2^	0.001** (0.000)	
**BMI (ref: normal weight)**		
Undernourished	−0.115 (0.244)	−0.115 (0.240)
Overweight	−0.114** (0.050)	−0.106** (0.049)
Obese	−0.268*** (0.055)	−0.250*** (0.054)
Difficulties moving around indoors	−0.340*** (0.108)	−0.322*** (0.105)
Number of difficulties in the activities of daily living	−0.127*** (0.022)	−0.139*** (0.021)
Number of difficulties in the instrumental activities of daily living	−0.032 (0.023)	
Depressed	−0.363*** (0.047)	−0.365*** (0.047)
Nervous	−0.209*** (0.044)	−0.206*** (0.043)
Lack of energy	−0.345*** (0.051)	−0.349*** (0.051)
Time awareness	0.069* (0.036)	0.069** (0.037)
**Marital status (ref: widowed)**		
Married	0.046 (0.074)	0.014 (0.055)
Single	−0.126 (0.109)	−0.094 (0.109)
Divorced/separated	0.229** (0.111)	0.210** (0.107)
Lives with someone else	−0.076 (0.077)	
Has someone to confide in	0.163** (0.077)	0.164* (0.076)
Years of education	0.064*** (0.018)	0.065*** (0.018)
Years of education^2^	−0.001 (0.001)	−0.001 (0.001)
Type of job: manual work	−0.152*** (0.045)	−0.147*** (0.044)
Vigorous physical activities	0.186*** (0.045)	0.199*** (0.045)
Moderate physical activities	0.306*** (0.044)	0.309*** (0.043)
**Smoking status (ref: non-smoker)**		
Former smoker	0.034 (0.055)	0.055 (0.051)
Current smoker	0.226*** (0.052)	0.241*** (0.051)
SHARE	0.408*** (0.050)	0.427*** (0.048)
Cutoff 1	−3.204** (1.268)	−0.309* (0.169)
Cutoff 2	−1.586 (1.267)	−1.303*** (1.171)
Observations	3,606	3,643
Pseudo *R*^2^	0.138	0.135

*Robust SEs in parentheses*.

*, **, and *** denote statistical significance at the 10, 5, and 1% significance levels.

We focus on the results of Model 2. The average marginal effects are shown in Table 2. For instance, on average and keeping all else constant, an obese person is seven percentage points less likely to report good, very good, or excellent health than a normal weight person. Having difficulties moving around indoors decreases the probability of reporting the highest level of health by nine percentage points. All three emotional status indicators have sizeable associations with self-assessed health. For example, feeling depressed decreases the likelihood of reporting good, very good, or excellent health by about ten percentage points. Having someone to confide in increases the probability of reporting the highest level of health by almost five percentage points. Engaging in moderate physical activities, such as going for a walk, contributes even more to the probability of reporting the highest level of health than engaging in more vigorous activities, such as sports (about nine and six percentage points, respectively). Taking into account the sample means and proportions, presented in Table S6 in Supplementary Material, all estimated associations are sizeable. Finally, on average individuals from the SHARE sample are more likely to report the highest level of health.

**Table 2:** Marginal effects in Model 2.

	Probability of reporting		
	Poor, bad, or very bad health	Fair health	Good, very good, or excellent health
	Average marginal effect (SE)	Average marginal effect (SE)	Average marginal effect (SE)
**BMI (ref: normal weight)**			
Undernourished	0.029 (0.060)	0.004 (0.008)	−0.032 (0.067)
Overweight	0.027** (0.012)	0.003** (0.002)	−0.030** (0.014)
Obese	0.062*** (0.014)	0.008*** (0.002)	−0.070*** (0.015)
Difficulties moving around indoors	0.080*** (0.026)	0.010** (0.004)	−0.091*** (0.029)
Number of difficulties in the activities of daily living	0.035*** (0.005)	0.004*** (0.001)	−0.039*** (0.006)
Depressed	0.091*** (0.012)	0.012*** (0.003)	−0.103*** (0.013)
Nervous	0.051*** (0.011)	0.007*** (0.002)	−0.058*** (0.012)
Lack of energy	0.087*** (0.013)	0.011*** (0.003)	−0.098*** (0.014)
Time awareness	−0.017* (0.009)	−0.002* (0.001)	0.019* (0.010)
**Marital status (ref: widowed)**			
Married	−0.003 (0.014)	−0.000 (0.002)	0.004 (0.016)
Single	0.023 (0.027)	0.003 (0.004)	−0.026 (0.031)
Divorced/separated	−0.052* (0.027)	−0.007* (0.004)	0.059* (0.030)
Has someone to confide in	−0.041** (0.019)	−0.005* (0.003)	0.046** (0.022)
Years of education	−0.016*** (0.004)	−0.002*** (0.001)	0.018*** (0.005)
Years of education^2^	0.000 (0.000)	0.000 (0.000)	−0.000 (0.000)
Type of job: manual work	0.037*** (0.011)	0.005*** (0.002)	−0.041*** (0.012)
Vigorous physical activities	−0.050*** (0.011)	−0.006*** (0.002)	0.056*** (0.013)
Moderate physical activities	−0.077*** (0.011)	−0.010*** (0.002)	0.087*** (0.012)
**Smoking status (ref: non-smoker)**			
Former smoker	−0.014 (0.013)	−0.002 (0.002)	0.015 (0.014)
Current smoker	−0.060*** (0.013)	−0.008*** (0.002)	0.068*** (0.014)
SHARE	−0.106*** (0.012)	−0.014*** (0.003)	0.120*** (0.013)

*Robust standard errors in parentheses*.

*, **, and *** denote statistical significance at the 10, 5, and 1% significance levels.

Including interactions between the MAB indicators and a binary indicator of the observation’s source (EPEPP or SHARE) produces the results presented in Table S7 in Supplementary Material. Out of the 20 interaction terms, only 4 are statistically significant, indicating that most associations between the MAB indicators and self-assessed health do not depend on the source of data. If allowed to differ according to the data source, the cutoffs are also not significantly different at the 5% significance level (results available upon request).

### Calculating the SAI

Selfie Aging Index scores for all individuals in the combined dataset are calculated based on Model 2. Figure S1 in Supplementary Material shows the distributions of the SAI according to selected individual characteristics. It is important to note that the distributions are unconditional, i.e., there is no adjustment for other characteristics.

### Validity Checks

Table S8 in Supplementary Material shows the results from regressions of the probability of having a doctor visit, number of doctor visits, mental health, number of chronic conditions, and number of symptoms on the SAI score. The associations between the SAI score and each outcome considered are negative, as expected. The *t*-statistics associated with the SAI coefficients are considerably high, indicating statistical significance at any conventional significance level. The *R*^2^s are also sizeable. These results reveal that the SAI is a strong predictor of the probability of having a doctor visit, number of doctor visits, mental health, number of chronic conditions, and number of symptoms.

## Discussion

### Findings from the Ordered Probit Models

In general, the MAB indicators have the expected associations with self-assessed health. There are only a few exceptions. In the EPEPP model, having complaints about hearing has a positive estimated coefficient, and spending less than 8 h per day alone has a negative estimated coefficient. Spending less than 8 h per day alone may be capturing other unmeasured physical or cognitive limitations that require the person to be accompanied most of the time. Globally, the two counterintuitive signs may be due to multicollinearity. For example, hearing and vision complaints are highly correlated, and spending less than 8 h per day alone is highly correlated with needing auxiliary instruments to perform the ADLs as well as living with someone else. High correlations between included variables affect the efficiency of the estimators, which may result in unexpected estimates. Also intriguingly, former and current smokers rate their health better than those who never smoked. Smoking status may also be capturing unobserved characteristics. For instance, people aged 55+ who still smoke possibly never had any serious health condition that would induce them to quit. Former smokers may have quit in time to avoid serious health damages. In the SHARE model, living with someone else has an estimated negative association with self-assessed health. The reasons for that may be the same ones mentioned above regarding the negative estimated coefficient associated with spending less than 8 h per day alone in the EPEPP model. For example, living with someone else is highly correlated with marital status, which may introduce significant multicollinearity in the model. Finally, in the SHARE model divorced individuals tend to have higher SAI scores than married individuals. Again, this finding may be due to multicollinearity or unobserved characteristics related to both health and selection into divorce (e.g., urban or rural area of residence, traditional values such as the idea that marriage is for life).

The MAB indicators that stand out in both the EPEPP and SHARE models are health complaints, difficulties performing the ADLs, felling depressed, feeling nervous, lacking energy, having someone to confide in, and exercising. This list includes indicators from all three domains of the MAB—biological, psychological, and social. Thus, a meaningful number of factors appear relevant in both models, providing external validity to our approach. It should be noted that lack of statistical significance of other MAB indicators may be attributable to the specific samples used or to lack of statistical power. It may also be the case that people simply do not consider some factors when asked to assess their health status. For instance, when self-evaluating their health status, people may be more likely to consider fear of falling, which is not measured here, than past falls, which is measured but not statistically significant.

Combining the two datasets allowed for improvements in statistical power, as the sample size increased substantially. It also implied two main drawbacks. First, several variables, including self-assessed health, had to be recoded. Still, our explorations suggest that this doesn’t represent a significant problem. Second, we had to exclude health complaints. Health complaints appeared relevant in both the EPEPP and SHARE models. Excluding this indicator remains a drawback to be addressed in future work.

Difficulties performing the IADLs and living with someone else, as well as age and gender, were excluded from the final model based on statistical criteria. Conceptually and given our experience, there are some reasons why these variables may appear as not relevant for self-assessed health. The IADLs include tasks that some people do not have the ability to perform or are used to request help with, such as managing money and using transportation. Unlike difficulties in the ADLs, difficulties in the IADLs do not necessarily capture disability. Living with someone else may loose importance in our model because related variables, such as marital status and having someone to confide in, are included. Besides, one may argue that it is the need to live with someone else rather than living with someone else or not that is relevant for self-assessed health. Such need is determined by the individual’s bad health and disability, which is captured by other variables in the model. Finally, age and gender may affect individuals’ perception of their health status mainly due to external factors, e.g., social misconceptions such as the idea that older ages are necessarily associated with worse health. Once other biopsychosocial factors are taken into account, it seems plausible that age and gender should no longer play a role.

The individual SAI scores calculated based on Model 2 have the expected distributions according to most individual characteristics. A few distributions may appear intriguing because they are unadjusted for other characteristics. For example, men tend to have higher SAI scores than women. This may be partly explained by the male–female health-survival paradox—the phenomenon that women experience greater longevity but higher rates of disability and poor health than men at more advanced ages [e.g., Ref. (21)]. As seen above, once other characteristics are taken into account, there are no significant differences in self-assessed health due to gender. As discussed above, smoking status and marital status may be capturing both observed and unobserved characteristics, which may explain the higher SAI scores among current and former smokers, as well as among divorced individuals. Finally, SAI scores tend to be higher in the SHARE sample than in the EPEPP sample. This may be partly related to the different socioeconomic environments that people faced in 2005–2006 and 2011 (before and after the financial crisis).

### The SAI in Practice

**Figure 1:**
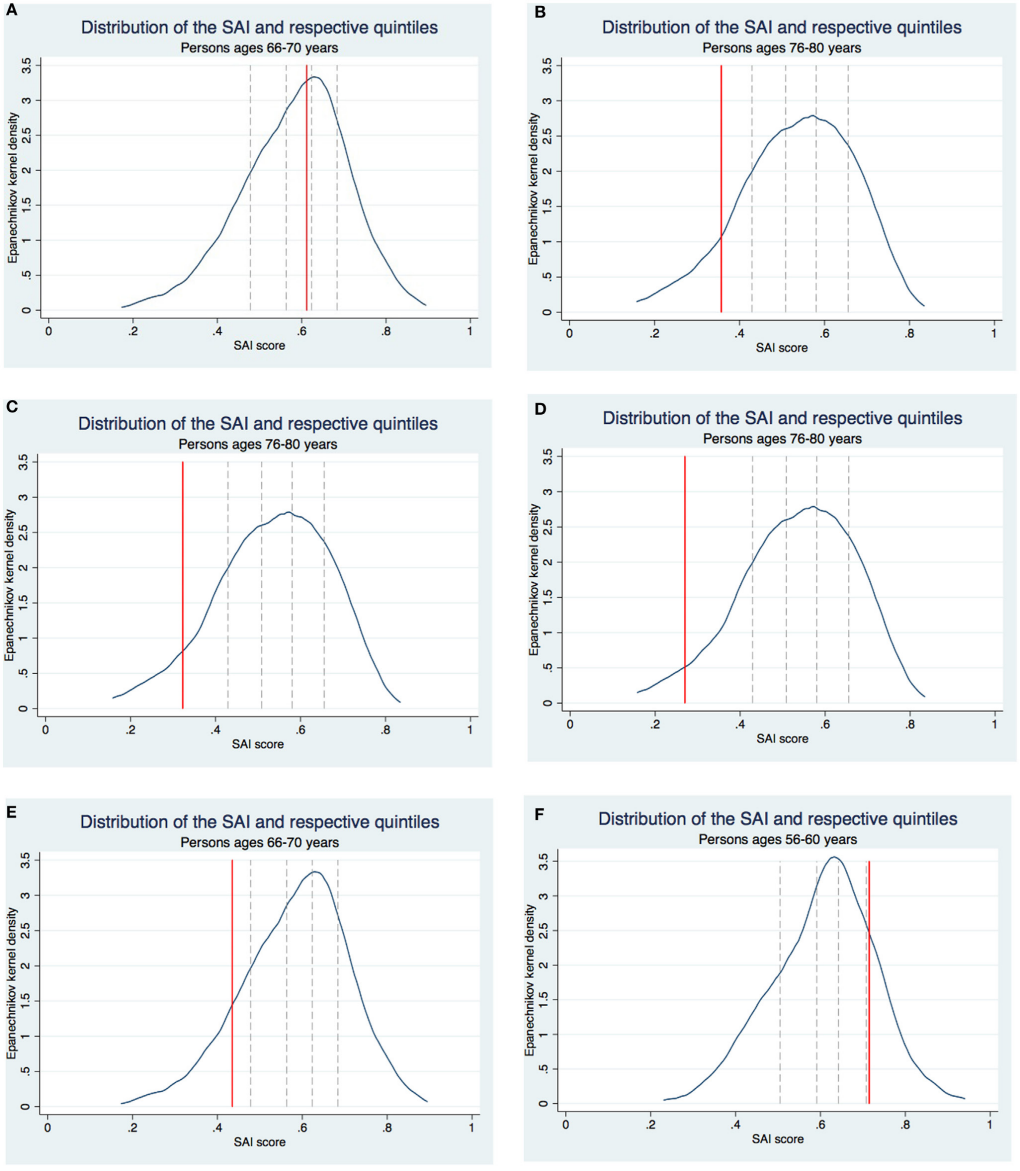

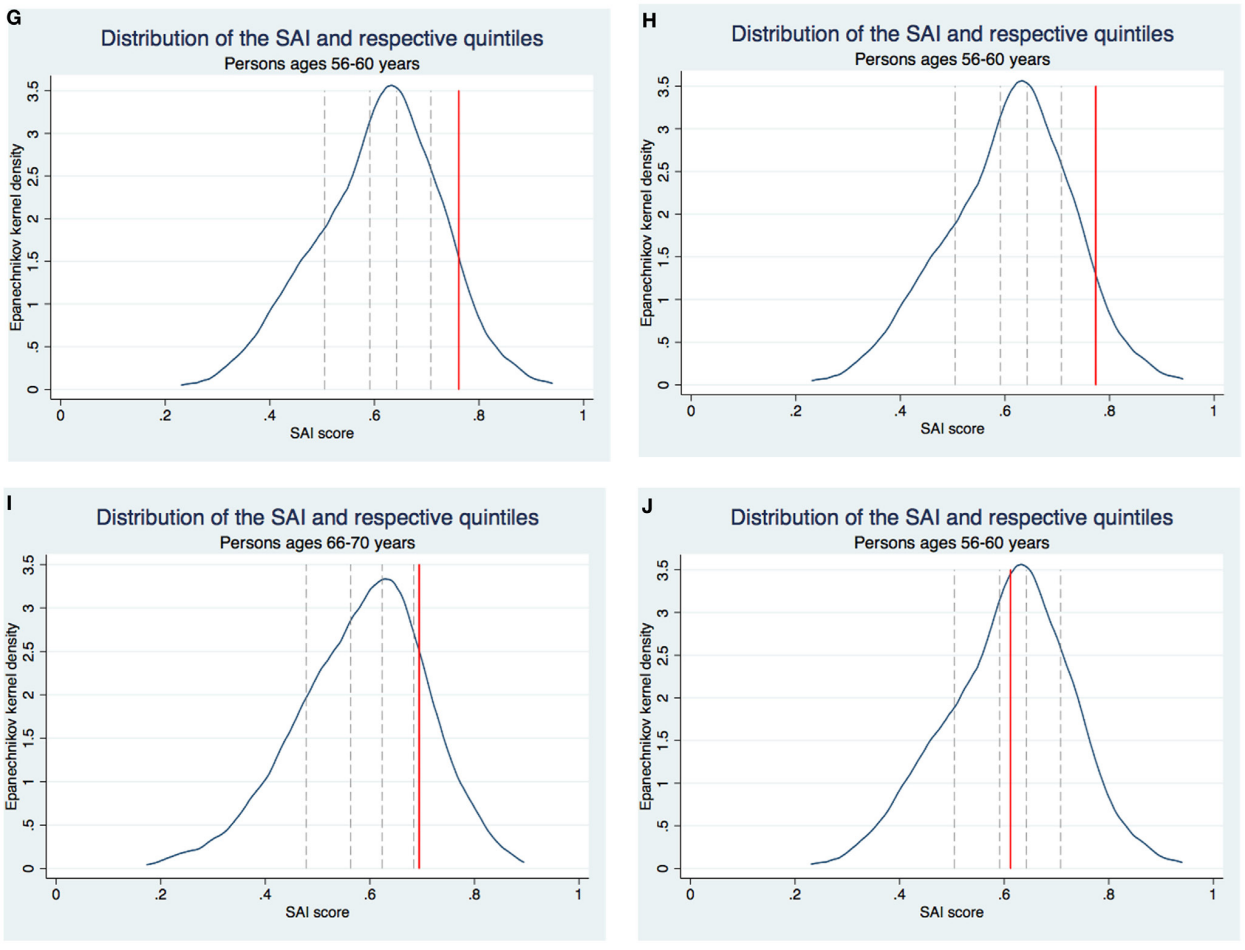
Selfie Aging Index (SAI) scores of the hypothetical individuals. **(A)** Mary. **(B)** James. **(C)** Margaret. **(D)** John. **(E)** Michael. **(F)** Linda. **(G)** Charles. **(H)** Susan. **(I)** Elisabeth. **(J)** William.

If the SAI is to be a useful tool for individuals to assess their aging status, in addition to an absolute value, it should have a relative component. The absolute value allows monitoring one’s aging status over time, while the relative component allows individuals to position themselves with respect to their peers. Thus, to illustrate how a tool based on the SAI might look like in practice, we consider ten hypothetical individuals, characterized in Table 3. The individuals’ characteristics are chosen to illustrate how the SAI changes as a result of changes in the various selected indicators. For each individual, we calculate his or her SAI score, and present it along with the distribution of the SAI among his or her peers (people aged ±2 years in the sample) in Figure 1.

**Table 3:** Characteristics of the hypothetical individuals.

Panel (A)					
	Mary	James	Margaret	John	Michael
Age	68	**78**	**78**	**78**	68
BMI	Overweight	Overweight	Overweight	Overweight	Overweight
Difficulties moving around indoors	No	**Yes**	**Yes**	**Yes**	No
Number of difficulties in the activities of daily living (ADLs)	Zero	**Five**	**Five**	**Five**	Zero
Depressed	No	No	No	No	**Yes**
Nervous	No	No	No	No	**Yes**
Lack of energy	No	No	No	No	**Yes**
Time awareness	Four	Four	Four	**Zero**	Four
Marital status	Married	Married	**Widowed**	**Widowed**	Married
Has someone to confide in	Yes	Yes	**No**	**No**	Yes
Years of education	Four	Four	Four	Four	Four
Type of job: manual work	Yes	Yes	Yes	Yes	Yes
Vigorous physical activities	No	No	No	No	No
Moderate physical activities	Yes	**No**	**No**	**No**	Yes
Smoking status	Non-smoker	Non-smoker	Non-smoker	Non-smoker	Non-smoker
Database	EPEPP	EPEPP	EPEPP	EPEPP	EPEPP
Selfie Aging Index (SAI) score	0.61	0.36	0.32	0.27	0.44
Quintile (compared to individuals within ±2 years of age)	3rd	1st	1st	1st	1st

**Panel (B)**					

	**Linda**	**Charles**	**Susan**	**Elisabeth**	**William**

Age	**58**	**58**	**58**	68	**58**
BMI	Overweight	Overweight	**Normal weight**	Overweight	Overweight
Difficulties moving around indoors	No	No	No	No	No
Number of difficulties in the ADLs	Zero	Zero	Zero	Zero	Zero
Depressed	No	No	No	No	No
Nervous	No	No	No	No	No
Lack of energy	No	No	No	No	No
Time awareness	Four	Four	Four	Four	Four
Marital status	Married	Married	Married	Married	Married
Has someone to confide in	Yes	Yes	Yes	Yes	Yes
Years of education	**Twelve**	**Twelve**	**Twelve**	Four	Four
Type of job: manual work	**No**	**No**	**No**	Yes	Yes
Vigorous physical activities	No	No	**Yes**	No	No
Moderate physical activities	Yes	Yes	Yes	Yes	Yes
Smoking status	Non-smoker	**Smoker**	Non-smoker	Non-smoker	Non-smoker
Database	EPEPP	EPEPP	EPEPP	**SHARE**	EPEPP
SAI score	0.71	0.76	0.77	0.69	0.61
Quintile (compared to individuals within ±2 years of age)	5th	5th	5th	5th	3rd

*The characteristics that differ from those of Mary are in bold*.

Consider for example Mary, a 68-year-old overweight woman. She has no difficulties moving around indoors or performing the ADLs. She is not depressed or nervous, and does not lack energy. She has a perfect score in time awareness. She is married and has someone to confide in. She has 4 years of education and had a manual job prior to retirement. She engages in moderate physical activities but not in more vigorous ones and does not smoke. Mary has a SAI score of 0.61, which puts her in the middle quintile of the distribution of the SAI among her peers (Figure 1A).

Take a second example, Michael, who is just like Mary except he is not in good shape emotionally. He has a SAI score of 0.44, which puts him in the bottom quintile of the distribution of the SAI among his peers (Figure 1E). Besides comparing individuals with different characteristics, the SAI can be used to infer what would happen if someone’s characteristics changed. In this example, Michael’s SAI score represents what we might expect of Mary’s if her emotional status would deteriorate.

As a final example, consider William. He has the same characteristics as Mary except he is 58 instead of 68. Given that age is not included in the SAI’s calculation, William and Mary’s scores are equal, but they compare themselves to different reference groups. Mary’s peers are individuals aged 66–70 and William’s are individuals aged 56–60. As the SAI distributions in the two age groups are very similar, in the end both William and Mary are in the middle quintile of the distributions of the SAI among their respective peers (Figures 1A,J).

## Conclusion

Our results shed light on various factors that contribute significantly to healthy and active aging. Two examples are mental health and exercise, which deserve more attention from individuals themselves, health-care professionals, and public health policy.

This study provides several lines for future research. Other methods to construct multidimensional indices should be tested and results should be compared with the ones presented here. Our methodology can be applied to data from other countries, to compare healthy and active aging across countries. The effectiveness of the SAI as a tool to promote healthy and active aging at the individual and aggregate levels should be investigated.

This study develops the SAI to measure healthy and active aging at the individual level. It is designed thinking about its possible implementation as a tool for individuals to monitor their aging status. For this reason, it is completely based on self-assessed indicators. The goal is that the SAI allows each person to take a *selfie* of her aging status without requiring a health-care professional to operate the camera. We illustrate how the SAI may be implemented in practice and provide it with a relative component, whereby individuals can compare themselves to their peers. The SAI also has promising applications for health-care professionals. Though it does not replace the clinical assessment of health problems, it may motivate health-care professionals to adopt a more encompassing view of individuals’ health and aging status. The SAI is useful to inform the design of public health policies targeting the most vulnerable groups. In conclusion, the SAI has the potential to put the individual at the center of the healthy and active aging discussion, contribute to patient empowerment and promote patient-centered care. It can become a useful instrument to monitor healthy and active aging for different actors, including individuals themselves, health-care professionals, and policy makers.

## Author Contributions

JG wrote the manuscript, and all remaining authors revised it critically.

## Conflict of Interest Statement

The authors declare that the research was conducted in the absence of any commercial or financial relationships that could be construed as a potential conflict of interest.

## Footnotes

^1^Ryser V-A, Weaver F, and Gonçalves J (Forthcoming). Health-related inequalities in life satisfaction among the 50+ population in Europe: evidence from SHARE. *Swiss J Sociol*.

^2^Co-authors of this study are involved in the EPEPP study and the SHARE project in Portugal and have thorough knowledge of the datasets.

^3^The likelihood ratio test is a statistical test used to compare the goodness of fit of two nested models. Under the null hypothesis, the simpler model is better, because it fits the data as well as the more complex one, but is more parsimonious. Lower values of the AIC and BIC indicate better model fit.

## References

[1] Bousquet J Kuh D Bewick M Standberg T Farrell J Pengelly R Operational definition of active and healthy ageing (AHA): a conceptual framework. J Nutr Health Aging (2015) 19(9):955–60. 10.1007/s12603-015-0589-6 26482699 PMC12878898

[2] UNECE/EC. Active Ageing Index 2014: Analytical Report. Report prepared by Asghar Zaidi of Center for Research on Ageing, University of Southampton and David Stanton, under contract with United Nations Economic Commission for Europe (Geneva), co-funded by European Commission’s Directorate General for Employment, Social Affairs and Inclusion, Brussels (2015).

[3] Zaidi A Gasior K Hofmarcher M Lelkes O Marin B Rodrigues R Active Ageing Index 2012 – Concept, Methodology and Final Results. European Centre for Social Welfare Policy and Research, Vienna (2013). Available from: http://www.euro.centre.org/data/1453740620_84975.pdf

[4] AARP. Surveying the Ageing Landscape. (2017). Available from: http://journal.aarpinternational.org/a/b/2014/03/surveying-the-aging-landscape

[5] HelpAge. Global AgeWatch Index 2015. (2017). Available from: http://www.helpage.org/global-agewatch/

[6] Barros PP Almeida SV How relevant is active ageing? Evidence from Portugal. In: Zaidi A Harper S Howse K Lamura G Perek-Bialas J, editors. Building Evidence for Active Ageing Policies: Active Ageing Index and Its Potential. Palgrave McMillan (2015).

[7] Barslund M von Werder M Zaidi A Inequality in active ageing: evidence from a new individual-level index for European countries. Ageing Soc (2017). 10.1017/S0144686X17001052

[8] Bowling A. Enhancing later life: how older people perceive active ageing? Aging Ment Health (2008) 12(3):293–301. 10.1080/13607860802120979 18728941

[9] Kusumastuti S Derks MGM Tellier S di Nucci E Lund R Mortensen EL Successful ageing: a study of the literature using citation network analysis. Maturitas (2016) 93:4–12. 10.1016/j.maturitas.2016.04.010 27156006

[10] Lara J Godfrey A Evans E Heaven B Brown LJE Barron E Towards measurement of the Healthy Ageing Phenotype in life-style based intervention studies. Maturitas (2013) 76:189–99. 10.1016/j.maturitas.2013.07.007 23932426

[11] Kuh D Karunananthan S Bergman H Cooper R. A life-course approach to healthy ageing: maintaining physical capability. Proc Nutr Soc (2014) 73:237–48. 10.1017/S0029665113003923 24456831 PMC3981474

[12] Botelho MA Autonomia Funcional em Idosos: Caracterização multidimensional em idosos utentes de um centro de saúde urbano [Seniors’ Functional Autonomy: Multidimensional Characterization of Elderly Users of an Urban Health Center]. 1st ed Porto, Portugal: Edições Bial (2000).

[13] Fontes A Botelho MA Fernandes A A biopsychosocial evaluation method and the International Classification of Functioning, Disability, and Health (ICF). Educ Gerontol (2014) 40(9):686–99. 10.1080/03601277.2011.559856

[14] Lindeboom M Kerkhofs M Health and work of the elderly: subjective health measures, reporting errors and endogeneity in the relationship between health and work. J Appl Econ (2009) 24:1024–46. 10.1002/jae.1077

[15] Sirven N Debrand T. Social participation and healthy ageing: an international comparison using SHARE data. Soc Sci Med (2008) 67:2017–26. 10.1016/j.socscimed.2008.09.056 18973973

[16] Steptoe A Wright C Kunz-Ebrecht SR Iliffe S Dispositional optimism and health behavior in community-dwelling older people: associations with healthy ageing. Br J Health Psychol (2006) 11:71–84. 10.1348/135910705X42850 16480556

[17] Mota-Pinto A Rodrigues V Botelho A Veríssimo MT Morais A Alves C A sociodemographic study of ageing in the Portuguese population: the EPEPP study. Arch Gerontol Geriatr (2011) 52:304–8. 10.1016/j.archger.2010.04.019 20510469

[18] Oliveira CR Rosa MS Mota-Pinto A Botelho MA Morais A Veríssimo MT Estudo do Perfil do Envelhecimento da População Portuguesa [Study of the Ageing Profiles of the Portuguese Population]. (2010). Available from: http://rihuc.huc.min-saude.pt/bitstream/10400.4/992/1/ACS%20EPEPP%20LIVRO.pdf

[19] Rodrigues V Mota-Pinto A de Sousa B Botelho MA Alves C de Oliveira CR. The aging profile of the Portuguese population: a principal component analysis. J Community Health (2014) 39(4):747–52. 10.1007/s10900-014-9821-2 24519178

[20] Malter F Börsch-Supan A SHARE Wave 4, Innovations and Methodology. (2013). Available from: http://www.share-project.org/fileadmin/pdf_documentation/Method_FRB_FINAL.pdf

[21] Alberts SC Archie EA Gesquiere LR Altmann J Vaupel JW Christensen K The male-female health-survival paradox: a comparative perspective on sex differences in ageing and mortality. In: Weinstein M Lane MA, editors. Sociality, Hierarchy, Health: Comparative Biodemography: A Collection of Papers. Washington, DC: The National Academies Press, National Research Council (2014). p. 339–64. Committee on Population, Division of Behavioral and Social Sciences and Education. 25254285

