# Supplementary material for: Selfie Aging Index: An Index for the Self-assessment of Healthy and Active Aging
Source: Front Med (Lausanne). 2017 Dec 22;4:236. doi: 10.3389/fmed.2017.00236 (PMC5744477; doi:10.3389/fmed.2017.00236)
Supplement: Supplementary file 1 [file Table_1.PDF]

Table S1. Link between the MAB, EPEPP, and SHARE

**MAB domains and subdomains**	**EPEPP question(s)**	**SHARE question(s)**
**Biological Domain**		
Complaints about one's health status<br>1. General, unspecific complaints	Over the past month, did you have any complaints about your health status and which?<br>*[open question, answers classified into complaints that affect or not mobility, complaints regarding eyesight, and complaints regarding hearing]*	PH010 For the past six months at least, have you been bothered by any of the health conditions on this card?<br>PH010_7 Falling down<br>PH010_8 Fear of falling down<br>PH010_12 Fatigue<br>PH010_97 Other symptoms, not yet mentioned
2. Complaints regarding the blood and hematopoietic organs		n.a.
3. Complaints about the digestive system		PH010_10 Stomach or intestine problems, including constipation, air, diarrhea
4. Complaints regarding eyesight		PH043 How good is your eyesight for seeing things at a distance, like recognizing a friend across the street?<br>PH044 How good is your eyesight for seeing things up close, like reading ordinary newspaper print?
5. Complaints regarding hearing		PH046 Is your hearing [*excellent/very good/good/fair/poor*]?
6. Complaints about the circulatory system		PH010_2 Heart trouble or angina, chest pain during exercise<br>PH010_5 Swollen legs
7. Complaints regarding the musculoskeletal system		PH010_1 Pain in your back, knees, hips or any other joint
8. Complaints about the nervous system		PH010_9 Dizziness, faints or blackouts
9. Complaints regarding the respiratory system		PH010_3 Breathlessness, difficulty breathing<br>PH010_4 Persistent cough
10. Complaints about the skin		n.a.
11. Complaints about the endocrinal system, metabolism, nutrition		n.a.
12. Complaints regarding the urinary system		PH010_11 Incontinence or involuntary loss of urine
13. Complaints about the reproductive system		n.a.
Nutrition status<br>1. BMI	Calculation based on height and weight measurements	Calculation based on height and weight measurements
2. Waist measure	Waist measurement	n.a.
Falls<br>1. Number of falls	If you fell in the past year, how many times did you fall?	n.a.
2. Moment of falls	Have you ever fallen? How long ago?	n.a.
3. Cause of falls	If you ever fell, what were the causes?	n.a.
4. Sequelae of falls	If you ever fell, do those falls still limit you in some way?	n.a.
Mobility<br>1. Moving around indoors	Over the past month, did you need help walking around the house?	PH049_2 Because of a health or memory problem, do you have difficultywalking across a room?

2. Moving around outdoors	Over the past month, did you need help walking outside?	n.a.
3. Climbing stairs	Over the past month, did you need help climbing stairs?	n.a.
Activities of daily living (ADLs)<br>1. Bathing	Over the past month, did you need help to wash/take a bath?	PH049 Because of a health or memory problem, do you have difficulty<br>PH049_3 Bathing or showering
2. Dressing	Over the past month, did you need help to get (un/)dressed?	PH049_1 Dressing, including putting on shoes and socks
3. Using the toilet	Over the past month, did you need help to use the toilet?	PH049_6 Using the toilet, including getting up or down
4. Getting in and out of bed	Over the past month, did you need help to lay down/get out of bed?	PH049_5 Getting in or out of bed
5. Sitting down and rising from chairs	Over the past month, did you need help to sit or rise from chairs?	n.a.
6. Bladder control	Over the past month, did you need help due to lack of bladder control?	n.a.
7. Bowel control	Over the past month, did you need help due to lack of bowel control?	n.a.
8. Eating	Over the past month, did you need help to eat?	PH049_4 Eating, such as cutting up your food
Instrumental activities of daily living (IADLs)<br>1. Using the phone	Over the past month, did you need help to use the phone?	PH049_10 Making telephone calls
2. Shopping	Over the past month, did you need help to shop?	PH049_9 Shopping for groceries
3. Preparing meals	Over the past month, did you need help to prepare your meals?	PH049_8 Preparing a hot meal
4. Housekeeping	Over the past month, did you need help to do your housekeeping?	PH049_12 Doing work around the house or garden
5. Doing the laundry	Over the past month, did you need help to do the laundry?	n.a.
6. Using transportation	Over the past month, did you need help to use transportation?	n.a.<br>*[Extra]* PH049_7 Using a map to figure out how to get around in a strange place
7. Taking medication	Over the past month, did you need help to take your medication?	PH049_11 Taking medications
8. Managing money	Over the past month, did you need help to manage your money?	PH049_13 Managing money, such as paying bills and keeping track of expenses
**Psychological domain**		
Emotional status<br>1. Complaints regarding one's emotional status	Over the past month, how do you classify your emotional status? *[good/very good, fair, poor, bad/very bad]*	MH022 Has a doctor ever told you that you suffer from other affective or emotional disorders, including anxiety, nervous or psychiatric problems?
2. Feelings of dismay/hopelessness	Over the past month, did you feel sad or depressed?	MH002 In the last month, have you been sad or depressed?
3. Lack of interest	n.a.	MH008 In the last month, what is your interest in things?<br>*[Less interest than usual mentioned / No mention of loss of interest/ Non-specific or uncodeable response]*

4. Feelings of nervousness/anxiousness	Over the past month, did you feel worried/nervous/anxious?	MH024 You are nervous… *[Never/ Hardly ever / Some of the time / Most of the time]*
5. Trouble sleeping	n.a.	MH007 Have you had trouble sleeping recently?
6. Lack of energy	Over the past month, did you lack energy?	MH013 In the last month, have you had too little energy to do the things you wanted to do?
Cognitive status – time awareness<br>1. Year	What year is this?	CF005 Which year is it?
2. Month	What month is this?	CF004 Which month is it?
3. Day of the month	What day of the month is this?	CF003 Which day of the month is it?
4. Season	What season is this?	n.a.
5. Day of the week	What day of the week is this?	CF006 Can you tell me what day of the week it is?
Cognitive status – spatial awareness<br>1. Country	What country is this?	n.a.
2. District	Which district do you live in?	n.a.
3. City	Which city do you live in?	n.a.
4. Building	Whose house is this?	n.a.
5. Floor	What floor is this?	n.a.
**Social domain**		
Social network<br>1. Marital status	What is your marital status?	DN014 What is your marital status?
2. Household size	Who do you live with?	Household size
3. Time alone in a 24-hour period	How many hours per day are you alone?	n.a.
4. Having someone to confide in	Do you have someone you can talk to about personal issues or ask for help?	Size of social network
Social status<br>1. Education	Number of years of education	Number of years of education
2. Type of job	What is/was your job?	EP016/EP052 Type of current or last job
Healthy behaviors<br>1. Exercising – walking	Over the past month, did you walk outside? How many hours per week?	n.a.
2. Exercising – doing sports	Over the past month, did you do any sports? How many hours per week?	BR015 How often do you engage in vigorous physical activity, such as sports, heavy housework, or a job that involves physical labor?
3. Exercising – other (e.g. housekeeping, gardening)	Over the past month, did you do other activities that require some physical effort? How many hours per week?	BR016 How often do you engage in activities that require a moderate level of energy such as gardening, cleaning the car, or doing a walk?
4. Smoking status	Do you smoke?	BR001/BR002 Have you ever smoked? Do you smoke at the present time?

Notes: n.a. = not available. For more details, see Fontes et al. (2014), Malter & Börsch-Supan (2013), and Oliveira et al. (2010).
