# Supplementary material for: Selfie Aging Index: An Index for the Self-assessment of Healthy and Active Aging
Source: Front Med (Lausanne). 2017 Dec 22;4:236. doi: 10.3389/fmed.2017.00236 (PMC5744477; doi:10.3389/fmed.2017.00236)
Supplement: Supplementary file 2 [file Table_2.PDF]

Table S2. Recoding of the variables matched between the EPEPP and SHARE

EPEPP question	SHARE question	New variable
**Over the past month, how do you classify your health?**	**Would you say your health is...**	**Self-assessed health (SAA)**
Bad/very bad	Poor	Poor/bad/very bad
Poor		
Fair	Fair	Fair
Good/very good	Good	Good/very good/excellent
	Very good	
	Excellent	
**Over the past month, did you need help walking around the house?**	**Because of a health or memory problem, do you have difficulty walking across a room?**	**Difficulties moving around indoors**
No, did so without any help	No	No
Yes, the help of auxiliary instruments	Yes	Yes
Yes, someone's help		
Can't walk around the house		
**Over the past month, did you need help to 1. wash, 2. get dressed, 3. use the toilet, 4. get out of bed, 5. to eat?**	**Because of a health or memory problem, do you have difficulty 1. bathing, 2. dressing, 3. using the toilet, 4. getting in or out of bed, 5. eating?**	**Difficulties in the ADLs**
No, did so without any help	No	No
Yes, the help of auxiliary instruments	Yes	Yes
Yes, someone's help		
Can't perform that activity		
**Over the past month, did you need help to 1. use the phone, 2. shop, 3. prepare your meals, 4. do your housekeeping, 5. take your medication, 6. manage your money?**	**Because of a health or memory problem, do you have difficulty 1. making calls, 2. shopping, 3. preparing a meal, 4. doing work around the house, 5. taking medications, 6. managing money?**	**Difficulties in the IADLs**
No, did so without any help	No	No
Yes, occasionally		
Yes, regularly	Yes	Yes
Can't perform that activity		
**Over the past month, did you feel sad or depressed?**	**In the last month, have you been sad or depressed?**	**Depressed**
No	No	No
Yes, little time		
Yes, half of the time	Yes	Yes
Yes, most of the time		
**Over the past month, did you feel worried/nervous/anxious?**	**You are nervous...**	**Nervous**
No	Never	No
Yes, little time	Hardly ever	
Yes, half of the time	Some of the time	Yes

Yes, most of the time	Most of the time	
**Over the past month, did you lack energy?**	**In the last month, have you had too little energy to do the things you wanted to do?**	**Lack of energy**
No	No	No
Yes, little time		
Yes, half of the time	Yes	Yes
Yes, most of the time		
**What is/was your job?**	**Type of current or last job**	**Type of job**
Manual work	Armed forces	Manual work
	Elementary	
	Blue collar	
	Crafts	
	Agriculture and fishing	
Specialized blue collar	Services and sales	Other non-manual work
Expert, sales	Clerk	
Technician	Technician	
	Professional	
Manager, army official	Manager, army official	
**Over the past month, did you do any sports?**	**How often do you engage in vigorous physical activity, such as sports, heavy housework, or a job that involves physical labor?**	**Vigorous physical activities**
Yes, more than 4 hours per week	More than once a week	At least once per week
Yes, 2-4 hours per week	Once a week	
Yes, less than 2 hours per week		
No	1-3 times per month	Less than once per week
	Hardly ever or never	
**Over the past month, did you walk outside?; Over the past month, did you do other activities that require some physical effort?**	**How often do you engage in activities that require a moderate level of energy, such as gardening, cleaning the car, or doing a walk?**	**Moderate physical activities**
Yes, more than 4 hours per week	More than once a week	More than once per week
Yes, 2-4 hours per week		
Yes, less than 2 hours per week	Once a week	Only once or less than once per week
No	1-3 times per month	
	Hardly ever or never	
**Do you smoke?**	**Have you ever smoked?; Do you smoke at the present time?**	**Smoking status**
Non-smoker	No	Non-smoker
Former smoker	Yes; No	Former smoker
Current smoker	Yes; Yes	Smoker
Occasional smoker or regularly exposed		
Former smoker and regularly exposed		

Notes: Only variables with questions posed differently in the two surveys.
