# Supplementary material for: Selfie Aging Index: An Index for the Self-assessment of Healthy and Active Aging
Source: Front Med (Lausanne). 2017 Dec 22;4:236. doi: 10.3389/fmed.2017.00236 (PMC5744477; doi:10.3389/fmed.2017.00236)
Supplement: Supplementary file 3 [file Table_3.PDF]

Table S3. Estimation results of the ordered probit model using the EPEPP sample

	Coefficient	Standard error
Gender: female	-0.056	(0.080)
Age	-0.016	(0.051)
Age^2	0.000	(0.000)
Complaints that affect mobility	-0.248***	(0.057)
Complaints that don't affect mobility	-0.331***	(0.054)
Complaints about eyesight	-0.140**	(0.068)
Complaints about hearing	0.158**	(0.071)
BMI (ref: normal weight)		
Undernourished	-0.052	(0.421)
Overweight	-0.020	(0.078)
Obese	-0.112	(0.092)
Waist measurement (ref: normal weight)		
Obesity I	-0.130	(0.095)
Obesity II	-0.218**	(0.103)
Number of falls	-0.015	(0.015)
Moment of last fall (ref: did not fall)		
Less than a month ago	-0.044	(0.133)
Between one month and one year ago	0.074	(0.089)
More than one year ago	0.058	(0.066)
Reason for falling (ref: did not fall, external cause)		
Internal and external causes	-0.207	(0.271)
Internal cause	-0.287***	(0.087)
Sequelae from falling (ref: did not fall, no sequelae)		
Sequelae that affect mobility	-0.187*	(0.102)
Sequelae that do not affect mobility	-0.009	(0.079)
Mobility: needs an auxiliary instrument	0.014	(0.040)
Mobility: needs someone's help	-0.140	(0.111)
ADLs: needs an auxiliary instrument	-0.029	(0.022)
ADLs: needs someone's help	-0.110*	(0.061)
IADLs: needs an auxiliary instrument	-0.029	(0.026)
IADLs: needs someone's help	-0.034	(0.022)
Self-assessment of emotional status (ref: good/very good)		
Fair	-0.715***	(0.070)
Poor	-1.203***	(0.104)
Bad/very bad	-1.618***	(0.206)
Feels depressed (ref: no)		
Little time	-0.096	(0.068)
Half of the time	-0.135	(0.088)
Most of the time	-0.425***	(0.119)
Feels nervous (ref: no)		
Little time	-0.147*	(0.077)
Half of the time	-0.181**	(0.086)
Most of the time	-0.097	(0.108)

Lack of energy (ref: no)		
Little time	-0.137**	(0.062)
Half of the time	-0.053	(0.087)
Most of the time	-0.086	(0.132)
Time awareness	0.056	(0.036)
Spatial awareness	-0.037	(0.045)
Marital status (ref: widowed)		
Divorced/separated	0.087	(0.153)
Single	-0.131	(0.135)
Married	-0.004	(0.095)
Lives with someone else	0.028	(0.101)
Less than 8 hours/day alone	-0.184***	(0.062)
Has someone to confide in	0.205**	(0.093)
Years of education	0.030	(0.026)
Years of education^2	-0.000	(0.002)
Type of job (ref: manual work)		
Specialized blue collar	0.052	(0.069)
Technician	0.131	(0.085)
Expert, sales	0.036	(0.102)
Manager, army official	0.057	(0.159)
None specified	-0.317	(0.332)
Physical activities: walking, sports, other (ref: does not exercise)		
Up to 2 hours a week of one of the activities	0.079	(0.205)
2 to 4 hours a week of one of the activities	0.274	(0.202)
More than 4 hours a week of one of the activities	0.483**	(0.200)
Smoking status (ref: non-smoker)		
Current smoker	0.374***	(0.128)
Occasional smoker or regularly exposed	0.151**	(0.074)
Former smoker	0.139*	(0.078)
Former smoker and regularly exposed	0.182	(0.175)
Cutoff 1	-3.235*	(1.769)
Cutoff 2	-1.976	(1.769)
Cutoff 3	-0.050	(1.768)
Observations	2083	
Pseudo R^2	0.193	

Notes: Age and education enter the model as second-degree polynomials, i.e. both a linear and a quadratic term are included. Obesity I is defined by having a waist measurement of 81-88cm if a woman and 95-102cm if a man, and obesity II is defined by having a waist measurement of more than 88cm if a woman and more than 102cm if a man. Robust standard errors in parentheses. *, **, and *** denote statistical significance at the 10%, 5%, and 1% significance levels.
