# Supplementary material for: Selfie Aging Index: An Index for the Self-assessment of Healthy and Active Aging
Source: Front Med (Lausanne). 2017 Dec 22;4:236. doi: 10.3389/fmed.2017.00236 (PMC5744477; doi:10.3389/fmed.2017.00236)
Supplement: Supplementary file 4 [file Table_4.PDF]

Table S4. Estimation results of the ordered probit model using the SHARE sample

	Coefficient	Standard error
Gender: female	0.158*	(0.083)
Age	-0.161***	(0.059)
Age^2	0.001***	(0.000)
Complaints: general	-0.213***	(0.073)
Complaints: digestive system	-0.190**	(0.091)
Complaints: eyesight	-0.163**	(0.069)
Complaints: hearing	-0.459***	(0.076)
Complaints: circulatory system	-0.424***	(0.092)
Complaints: musculoskeletal system	-0.314***	(0.074)
Complaints: nervous system	-0.212**	(0.097)
Complaints: respiratory system	-0.274***	(0.102)
Complaints: urinary system	0.123	(0.148)
BMI (ref: normal weight)		
Undernourished	-0.348	(0.287)
Overweight	-0.093	(0.075)
Obese	-0.089	(0.097)
Difficulties moving around indoors	-0.605**	(0.303)
Number of difficulties in the ADLs	-0.213***	(0.063)
Number of difficulties in the IADLs	-0.054	(0.052)
Complaints: emotional status	-0.084	(0.088)
Depressed	-0.230***	(0.078)
Lack of interest (ref: no loss of interest)		
Less interest than usual	0.038	(0.114)
Uncodeable answer	0.349**	(0.154)
Nervousness (ref: never)		
Seldom	0.003	(0.090)
Sometimes	-0.055	(0.087)
Often	-0.235*	(0.140)
Trouble sleeping	-0.267***	(0.079)
Lack of energy	-0.226***	(0.079)
Time awareness	-0.007	(0.080)
Marital status (ref: widowed)		
Married	0.285**	(0.138)
Single	-0.038	(0.191)
Divorced/separated	0.397**	(0.182)
Lives with someone else	-0.239*	(0.140)
Has someone to confide in	0.089***	(0.021)
Years of education	0.071**	(0.031)
Years of education^2	-0.002	(0.002)
Type of job (ref: manager, army official)		
Professional	-0.114	(0.170)
Technician	0.077	(0.142)
Clerk	0.017	(0.156)

Services and sales	-0.039	(0.168)
Agriculture and fishing	-0.071	(0.194)
Crafts	-0.084	(0.162)
Blue collar	-0.280	(0.217)
Elementary	-0.032	(0.155)
Armed forces	0.167	(0.286)
Vigorous physical activities (ref: more than once a week)		
Once a week	-0.067	(0.120)
1-3 times a month	-0.114	(0.121)
(Almost) never	-0.217**	(0.084)
Moderate physical activities (ref: more than once a week)		
Once a week	0.035	(0.091)
1-3 times a month	0.121	(0.111)
(Almost) never	-0.177*	(0.098)
Smoking status (ref: non-smoker)		
Former smoker	-0.079	(0.082)
Current smoker	0.192	(0.125)
Cutoff 1	-7.559***	(2.048)
Cutoff 2	-5.754***	(2.044)
Cutoff 3	-4.505**	(2.042)
Cutoff 4	-3.788*	(2.042)
Observations	1245	
Pseudo R^2	0.217	

Notes: Age and education enter the model as second-degree polynomials, i.e. both a linear and a quadratic term are included. Robust standard errors in parentheses. *, **, and *** denote statistical significance at the 10%, 5%, and 1% significance levels.
