# Supplementary material for: Selfie Aging Index: An Index for the Self-assessment of Healthy and Active Aging
Source: Front Med (Lausanne). 2017 Dec 22;4:236. doi: 10.3389/fmed.2017.00236 (PMC5744477; doi:10.3389/fmed.2017.00236)
Supplement: Supplementary file 5 [file Table_5.PDF]

**Table S5.** Contribution of each variable or group of variables to model fit

| | p-value of the likelihood ratio test | Akaike's information criterion | Bayesian information criterion |
|---|---|---|---|
| Education | 0.0000 | 7186.779 | 7211.541 |
| Lack of energy | 0.0000 | 6994.771 | 7025.723 |
| Number of difficulties in the ADLs | 0.0000 | 6859.771 | 6896.913 |
| Depressed | 0.0000 | 6781.36 | 6824.692 |
| SHARE | 0.0000 | 6707.296 | 6756.819 |
| Moderate physical activities | 0.0000 | 6624.007 | 6679.720 |
| Nervous | 0.0000 | 6602.614 | 6664.517 |
| Smoking status | 0.0000 | 6580.818 | 6655.102 |
| Vigorous physical activities | 0.0000 | 6561.577 | 6642.051 |
| BMI | 0.0001 | 6545.909 | 6644.954 |
| Difficulties moving around indoors | 0.0002 | 6533.688 | 6638.925 |
| Type of job | 0.0004 | 6523.189 | 6634.615 |
| Time awareness | 0.0196 | 6519.737 | 6637.354 |
| Has someone to confide in | 0.0582 | 6518.149 | 6641.956 |
| Marital status | 0.0562 | 6516.596 | 6658.974 |
| Age | 0.1025 | 6516.039 | 6670.798 |
| Gender | 0.2941 | 6516.939 | 6677.888 |
| Number of difficulties in the IADLs | 0.1406 | 6516.768 | 6683.907 |
| Lives with someone else | 0.3167 | 6517.765 | 6691.095 |

Notes: Variables progressively included according to their contribution to the log-likelihood. Variables shaded in gray are excluded from Model 2.
