# Supplementary material for: Selfie Aging Index: An Index for the Self-assessment of Healthy and Active Aging
Source: Front Med (Lausanne). 2017 Dec 22;4:236. doi: 10.3389/fmed.2017.00236 (PMC5744477; doi:10.3389/fmed.2017.00236)
Supplement: Supplementary file 6 [file Table_6.PDF]

frontiers

Table S6. Descriptive statistics by sample

	Combined sample	EPEPP	SHARE
Gender: female	0.54	0.56	0.49
Age[a]	67.57	67.94	66.85
	(8.20)	(8.13)	(8.30)
	[55; 99]	[55; 99]	[55; 95]
BMI:			
Undernourished	0.01	0.00	0.01
Normal (ref)	0.24	0.20	0.32
Overweight	0.45	0.46	0.45
Obese	0.30	0.34	0.22
Difficulties moving around indoors	0.05	0.07	0.03
ADLs:			
Difficulties bathing	0.14	0.18	0.05
Difficulties dressing	0.09	0.06	0.14
Difficulties using the toilet	0.13	0.18	0.04
Difficulties getting out of bed	0.16	0.20	0.07
Difficulties eating	0.05	0.06	0.03
Number of difficulties in the ADLs[a]	0.56	0.68	0.33
	(1.08)	(1.15)	(0.87)
	[0; 5]	[0; 5]	[0; 5]
Depressed	0.37	0.31	0.48
Nervous	0.47	0.46	0.50
Lack of energy	0.26	0.21	0.33
Time awareness[a]	3.74	3.69	3.83
	(0.61)	(0.65)	(0.52)
	[0; 4]	[0; 4]	[0; 4]
Marital status:			
Married	0.74	0.72	0.77
Single	0.04	0.05	0.04
Divorced/separated	0.05	0.04	0.07
Widowed (ref)	0.17	0.19	0.12
Has someone to confide in	0.94	0.92	0.98
Years of education[a]	5.39	5.05	6.05
	(3.84)	(3.62)	(4.16)
	[0; 23]	[0; 20]	[0; 23]
Type of job: manual work	0.49	0.46	0.54
Vigorous physical activities	0.27	0.22	0.35
Moderate physical activities	0.62	0.71	0.44
Smoking status:			
Non-smoker (ref)	0.59	0.57	0.65

Former smoker	0.22	0.19	0.26
Current smoker	0.19	0.24	0.09
Observations	3643	2392	1251

Notes: Only variables in Model 2 and demographics included. [a]Continuous variable with standard deviation in parentheses and minimum and
