# Supplementary material for: Selfie Aging Index: An Index for the Self-assessment of Healthy and Active Aging
Source: Front Med (Lausanne). 2017 Dec 22;4:236. doi: 10.3389/fmed.2017.00236 (PMC5744477; doi:10.3389/fmed.2017.00236)
Supplement: Supplementary file 7 [file Table_7.PDF]

Table S7. Estimation results of Model 2 with interactions between all variables and the sample indicator variable

	Coefficient	Standard error
BMI (ref: normal weight)		
Undernourished	-0.156	(0.273)
Overweight	-0.082	(0.064)
Obese	-0.210***	(0.068)
Difficulties moving around indoors	-0.300***	(0.112)
Number of difficulties in the ADLs	-0.082***	(0.023)
Depressed	-0.344***	(0.061)
Nervous	-0.238***	(0.054)
Lack of energy	-0.309***	(0.067)
Time awareness	0.074*	(0.042)
Marital status (ref: widowed)		
Married	-0.046	(0.063)
Single	-0.107	(0.125)
Divorced/separated	0.142	(0.138)
Has someone to confide in	0.220***	(0.083)
Years of education	0.032	(0.022)
Years of education^2	0.000	(0.001)
Type of job: manual work	-0.146***	(0.054)
Vigorous physical activities	0.160***	(0.057)
Moderate physical activities	0.421***	(0.054)
Smoking status (ref: non-smoker)		
Former smoker	0.203***	(0.065)
Current smoker	0.256***	(0.057)
SHARE	0.833*	(0.425)
**Interactions between SHARE and:**		
BMI (ref: normal weight)		
Undernourished	0.131	(0.446)
Overweight	-0.056	(0.102)
Obese	-0.117	(0.117)
Difficulties moving around indoors	-0.123	(0.312)
Number of difficulties in the ADLs	-0.328***	(0.061)
Depressed	-0.052	(0.096)
Nervous	0.043	(0.091)
Lack of energy	-0.016	(0.102)
Time awareness	-0.038	(0.091)
Marital status (ref: widowed)		
Married	0.206	(0.134)
Single	0.139	(0.245)
Divorced/separated	0.228	(0.231)

Has someone to confide in	-0.326	(0.241)
Years of education	0.084**	(0.038)
Years of education^2	-0.003	(0.002)
Type of job: manual work	-0.026	(0.097)
Vigorous physical activities	0.104	(0.094)
Moderate physical activities	-0.347***	(0.092)
Smoking status (ref: non-smoker)		
Former smoker	-0.361***	(0.107)
Current smoker	-0.096	(0.136)
Cutoff 1	-0.246	(0.197)
Cutoff 2	1.393***	(0.199)
Observations	3643	
Pseudo R^2	0.148	

Notes: Robust standard errors in parentheses. *, **, and *** denote statistical significance at the 10%, 5%, and 1% significance levels.
