# Supplementary material for: Selfie Aging Index: An Index for the Self-assessment of Healthy and Active Aging
Source: Front Med (Lausanne). 2017 Dec 22;4:236. doi: 10.3389/fmed.2017.00236 (PMC5744477; doi:10.3389/fmed.2017.00236)
Supplement: Supplementary file 8 [file Table_8.PDF]

Table S8. Value of the SAI as a predictor of different outcomes

	Prob(doctor visit)	Ln(doctor visits)	EURO-D	Chronic conditions	Symptoms
SAI score	-2.226***	-1.869***	-14.181***	-5.716***	-10.521***
	(-7.223)	(-18.001)	(-33.854)	(-15.088)	(-21.160)
Intercept	2.901***	2.419***	12.115***	5.668***	8.996***
	(14.941)	(39.732)	(43.042)	(22.640)	(26.539)
Observations	3586	3372	1240	1251	1251
R^2	0.038	0.085	0.474	0.171	0.307

Notes: Robust t-statistics in parentheses. *** denotes statistical significance at the 0.1% significance level. All outcomes are estimated using linear regression except for the probability of having a doctor visit. In this case, a probit model is estimated and the pseudo-R^2 is reported.
