# Supplementary material for: Selfie Aging Index: An Index for the Self-assessment of Healthy and Active Aging
Source: Front Med (Lausanne). 2017 Dec 22;4:236. doi: 10.3389/fmed.2017.00236 (PMC5744477; doi:10.3389/fmed.2017.00236)
Supplement: Supplementary file 9 [file Image_1.PDF]

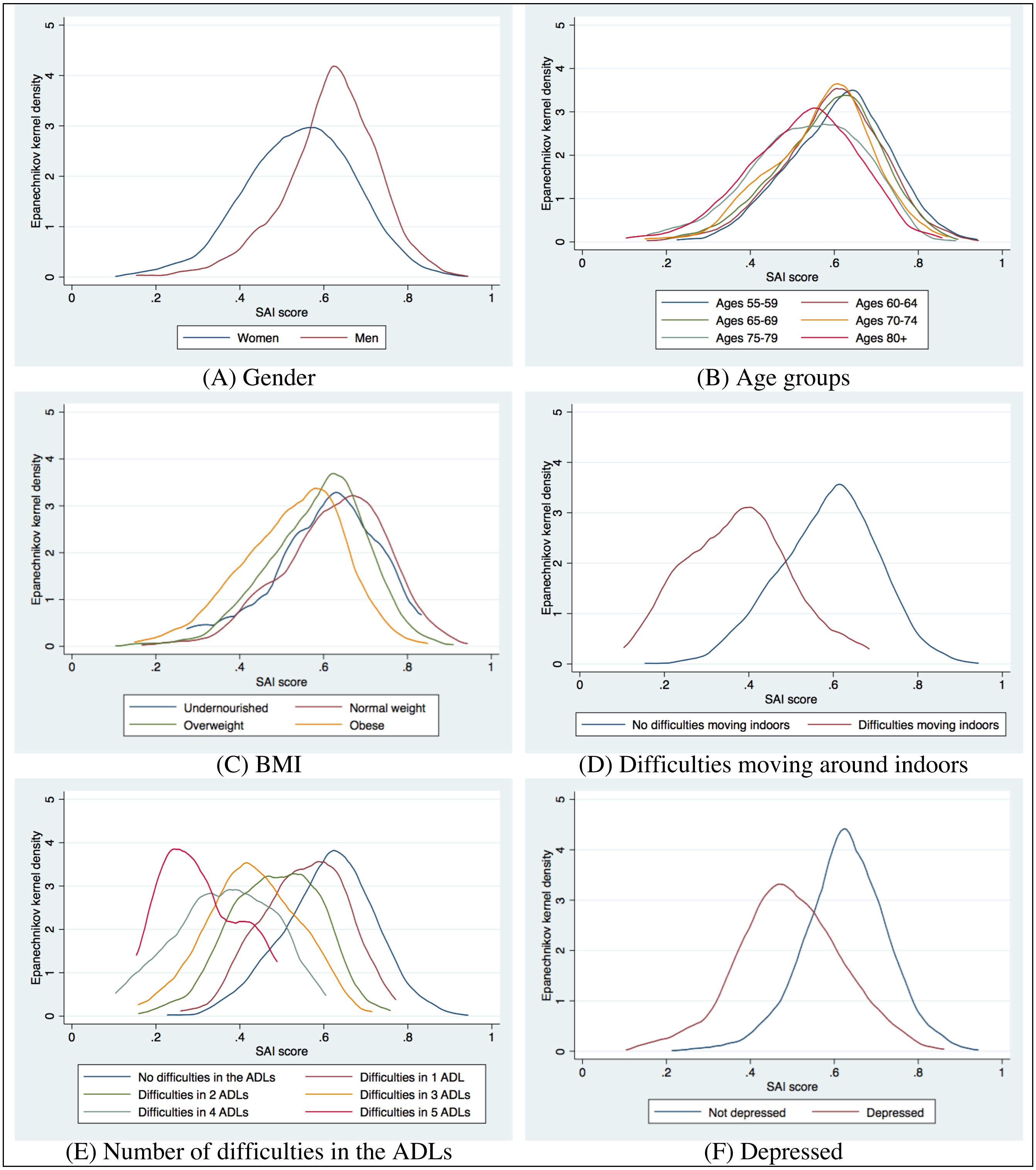
Epanechnikov kernel density
SAI score
Women
Men
(A) Gender
Ages 55-59
Ages 60-64
Ages 65-69
Ages 70-74
Ages 75-79
Ages 80+
(B) Age groups
Undernourished
Normal weight
Overweight
Obese
(C) BMI
No difficulties moving indoors
Difficulties moving indoors
(D) Difficulties moving around indoors
No difficulties in the ADLs
Difficulties in 1 ADL
Difficulties in 2 ADLs
Difficulties in 3 ADLs
Difficulties in 4 ADLs
Difficulties in 5 ADLs
(E) Number of difficulties in the ADLs
Not depressed
Depressed
(F) Depressed

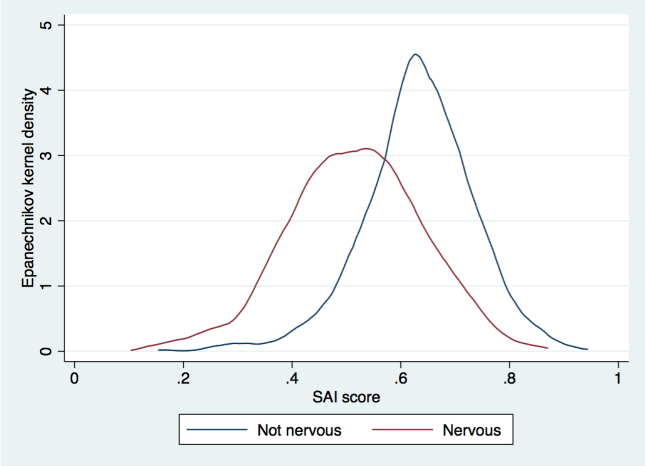

(G) Nervous

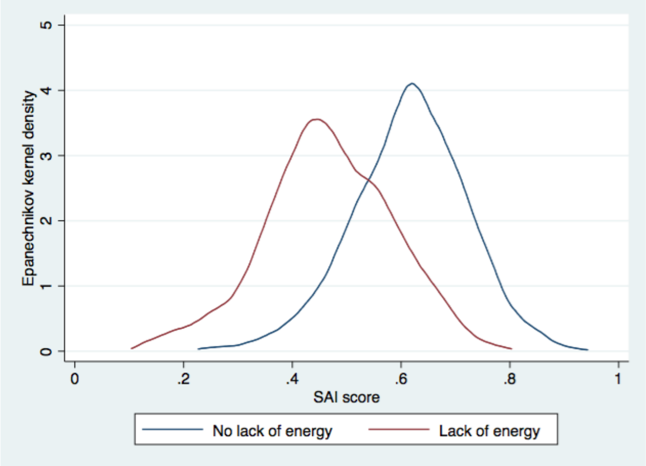

(H) Lack of energy

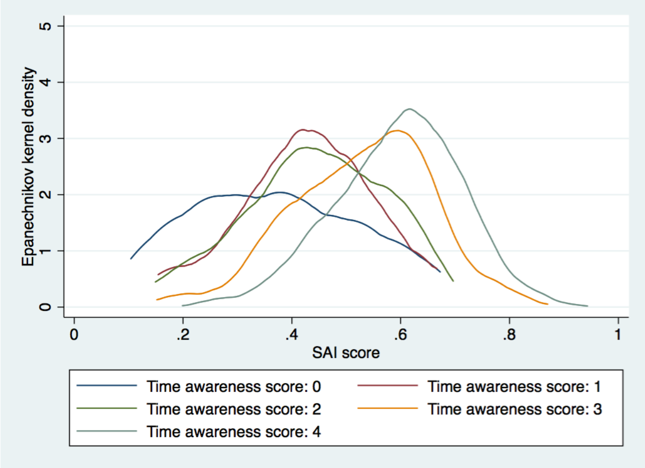

(I) Time awareness score

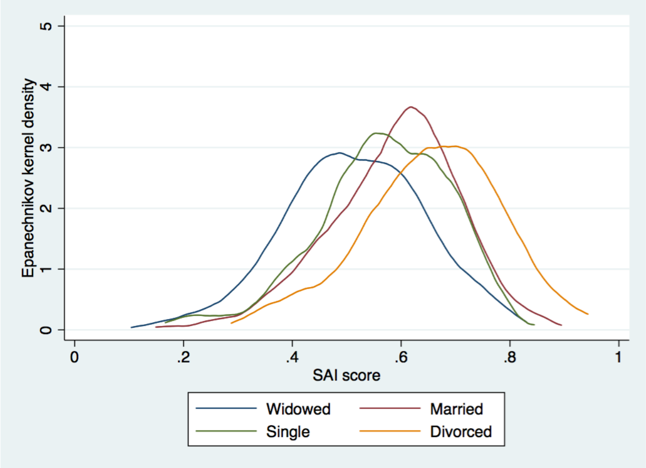

(J) Marital status

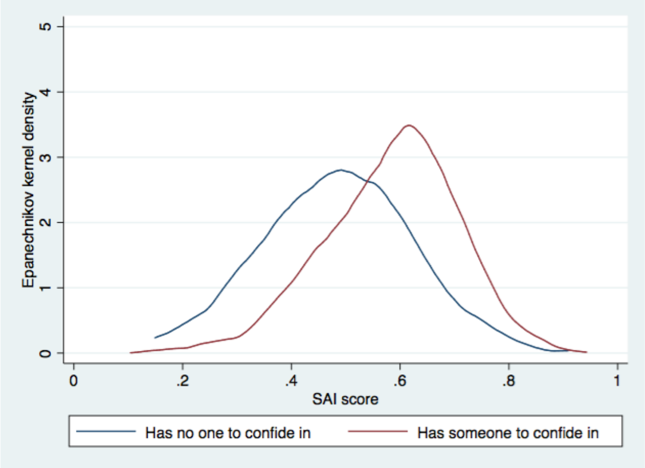

(K) Having someone to confide in

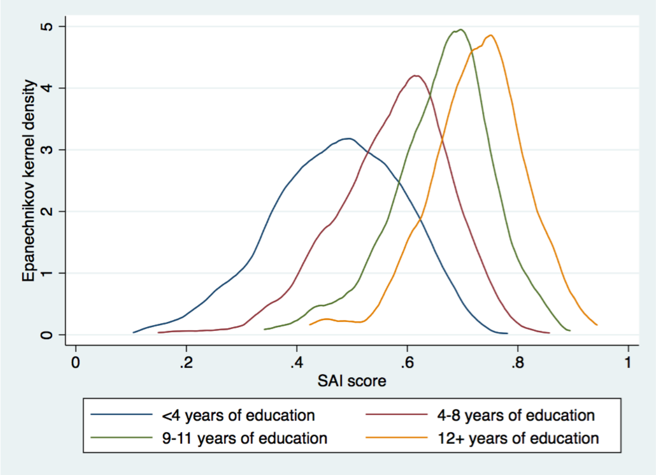

(L) Years of education

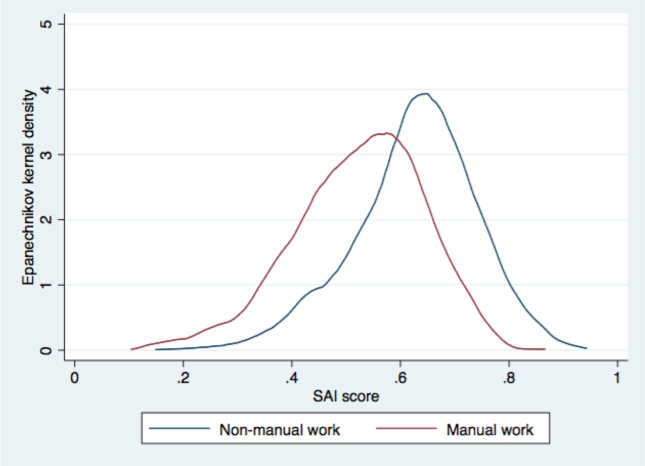

(M) Type of job

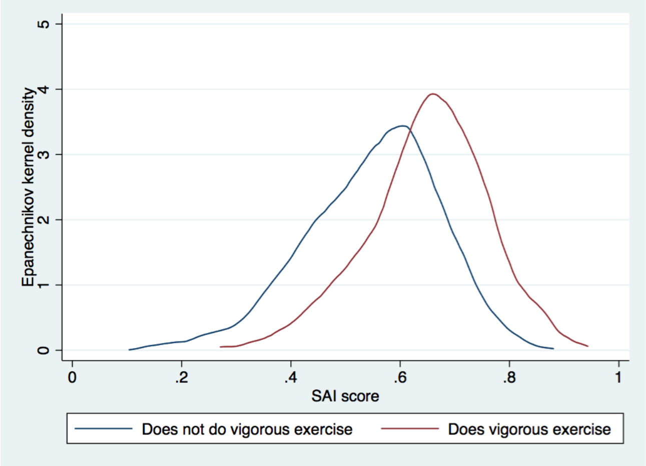

(N) Vigorous physical activities

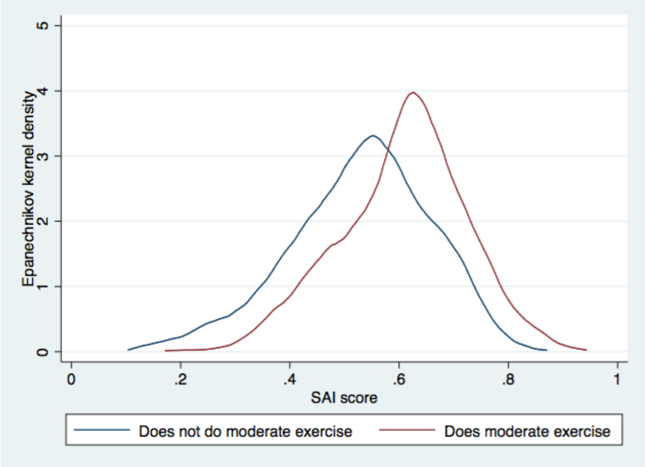

(O) Moderate physical activities

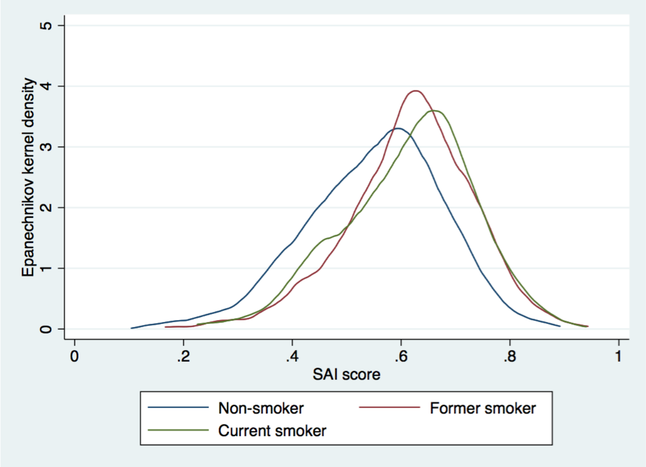

(P) Smoking status

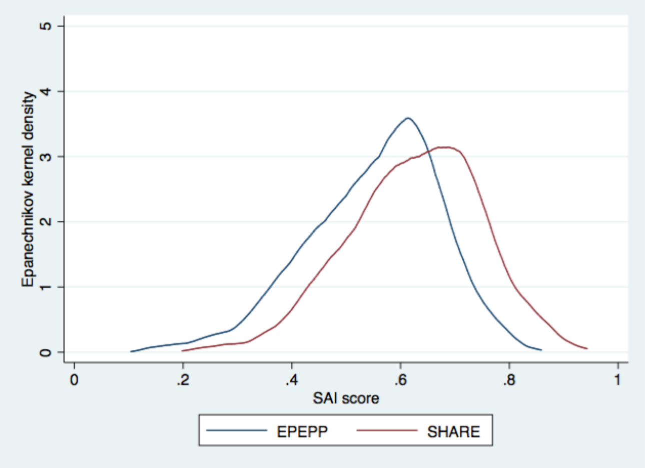

(Q) Sample

Figure S1. Distributions of the SAI according to selected characteristics
